# Characterization of a Genetic Variant in *BARD1* in Subjects Undergoing Germline Testing for Hereditary Tumors

**DOI:** 10.3390/biomedicines13112764

**Published:** 2025-11-12

**Authors:** Elena Marino, Elena Belloni, Matteo Dal Molin, Monica Marabelli, Aliana Guerrieri-Gonzaga, Cristina Zanzottera, Sara Mannucci, Mariarosaria Calvello, Francesca Fava, Irene Feroce, Bernardo Bonanni, Loris Bernard, Massimo Barberis, Pier Giuseppe Pelicci, Francesco Bertolini

**Affiliations:** 1 Laboratory of Medical Genetics, Cytogenetics and Molecular Genetics, IEO, European Institute of Oncology, IRCCS, 20141 Milan, Italy; matteo.dalmolin@ieo.it (M.D.M.); francesco.bertolini@ieo.it (F.B.); 2 Department of Experimental Oncology, IEO, European Institute of Oncology, IRCCS, 20141 Milan, Italy; elena.belloni@ieo.it (E.B.); massimo.barberis@ieo.it (M.B.); piergiuseppe.pelicci@ieo.it (P.G.P.); 3 Division of Cancer Prevention and Genetics, IEO, European Institute of Oncology, IRCCS, 20141 Milan, Italy; monica.marabelli@ieo.it (M.M.); aliana.guerrierigonzaga@ieo.it (A.G.-G.); sara.mannucci@ieo.it (S.M.); mariarosaria.calvello@ieo.it (M.C.); francesca.fava@ieo.it (F.F.); irene.feroce@ieo.it (I.F.); bernardo.bonanni@ieo.it (B.B.); 4 Department of Oncology and Haemato-Oncology, University of Milan, 20122 Milan, Italy; 5 Laboratory of Hematology-Oncology, IEO, European Institute of Oncology, IRCCS, 20141 Milan, Italy

**Keywords:** *BARD1*, HBOC, NGS

## Abstract

Hereditary breast and ovarian cancer (HBOC) syndrome accounts for 5–10% of all breast and ovarian cancers, with *BRCA1* and *BRCA2* pathogenic variants being the most common genetic alterations. However, additional genes such as *BARD1*, whose protein product interacts with BRCA1 via its N-terminal RING domain, have been implicated as low-penetrance contributors to cancer risk. This study aimed to investigate the frequency and distribution of the *BARD1* variant c.1518_1519delinsCA (p.Val507Met) in a cohort of 920 patients undergoing genetic testing for hereditary cancer predisposition. Next Generation Sequencing (NGS) was performed using a 28-gene panel, and allelic frequencies of *BARD1* were analyzed. Among 920 patients, 159 (17.28%) were pure heterozygous for the c.1518_1519delinsCA variant. Notably, c.1519G>A was never observed without c.1518T>C, suggesting a strong linkage between the two variants. The allele frequencies observed (34.51% for A at c.1519 and 77.88% for C at c.1518) challenge current reference genome expectations. Data from the ALFA database confirmed that these frequencies are consistent with population-level variation, not sample bias. Our findings raise the hypothesis that the reference allele at position c.1518 may not reflect the true wild-type sequence. While both c.1518T>C and c.1519G>A are individually classified as benign, their combined occurrence as a dinucleotide substitution (c.1518_1519delinsCA) warrants further investigation. These results underscore the importance of accurate variant annotation and population-specific frequency data for clinical interpretation of NGS findings. Although *BARD1* remains a low-frequency contributor to HBOC compared to *BRCA1/2*, its inclusion in multigene panels is supported by the potential relevance of such complex variants.

## 1. Introduction

About 5–10% of breast cancers (BCs) and approximately 20–25% of ovarian cancers (OCs) are due to germline pathogenic/likely pathogenic variants (PVs) in cancer predisposition genes, collectively referred to as Hereditary Breast and Ovarian Cancer (HBOC) syndrome. The *BRCA1* and *BRCA2* (*BRCA*) genes are the most frequently involved in HBOC. PVs in these genes are considered to be responsible for approximately 40% of familial BCs and for the majority of familial OCs [1]. However, to date, other cancer predisposition genes have been described as conferring an increased risk for BCs and/or OCs [2].

The BRCA1-associated RING domain 1 (*BARD1*) gene was originally identified because its encoded product interacts with the BRCA1 protein. Several PVs in *BARD1* have been reported both in non-*BRCA* HBOC patients and in patients with sporadic tumors [3]. *BARD1* is located on 2q34–35 and encodes a protein of 777 amino acids [4]. It was discovered in 1996, in an effort to elucidate the biological function of the BRCA1 protein, and it is considered a putative tumor suppressor that can form stable heterodimers with BRCA1. Indeed, it directly interacts with BRCA1 via its N-terminal RING domain. As *BRCA1* PVs located in the RING domain disrupt the BARD1-BRCA1 interaction, it was hypothesized that variants in *BARD1* may also affect this interaction, thereby making this gene an attractive candidate to investigate in BRCAX families (i.e., with no PVs in the *BRCA* genes) [5].

Thai et al. [6] were the first to describe a missense variant in *BARD1*, c.1692G>C (p.Gln564His). This germline variant was identified in a woman affected by a clear cell ovarian tumor, and loss of the wild-type *BARD1* allele was observed in the malignant cells. The variant is predicted to replace an amino acid residue located near the BRCT domains of BARD1, and this change may affect the normal function of the protein. Moreover, this patient was also diagnosed with two other primary cancers. The authors hypothesized a possible role for *BARD1* variants in the development of sporadic and hereditary tumors. [6].

Later, other PVs have been described in the literature, in the RING, ANK, and BRCT domains [5]. Currently, the *BARD1* PV frequency in HBOCs is less than 1% [3], compared to 30.4% and 28.6% for *BRCA1* and *BRCA2* genes, respectively. For this reason, along with other genes such as *ATM*, *CHEK2*, *RAD51C*, *RAD51D*, and *NF1*, it is considered a moderate-penetrance BC gene. Consequently, multigene panel testing often includes *BARD1* amongst the analyzed genes.

In recent years, some laboratories have investigated specific *BARD1* variants in order to assess their involvement in tumor development. The three most studied variants are c.70C>T (p.Pro24Ser), c.1134G>C (p.Arg378Ser), and c.1519G>A (p.Val507Met) [7,8,9], all classified as benign. By performing a case/control study in Japanese patients, Ishitobi et al. observed an association between the c.1519G>A variant and an increased risk of BC in post-menopausal women [10]. Huo et al. [7], in a case/control study in Chinese patients based on haplotype analysis, showed that people with the CT and TT genotype of the c.70C>T variant and the CC genotype of the c.1134G>C variant have a lower risk of BC as compared to wild-type (WT) homozygote subjects. Interestingly, a Spanish study [11] used two in tandem *BARD1* variants (c.1518T>C and c.1519G>A) to validate the tSNPh assay (tandem SNPs haplotyping assay), a method of haplotype analysis.

The classification of genomic variants according to their clinical significance ranges from benign to pathogenic, in accordance with the guidelines implemented by the American College of Medical Genetics and Genomics (ACMG) [12]. Assigning a given variant to one of the five defined categories (benign, likely benign, uncertain significance, likely pathogenic, pathogenic) is crucial for clinical decision-making. Notably, even before the clinical interpretation and classification of genetic variants, accurate annotation represents a crucial step in the NGS workflow.

In this study, we focused on two *BARD1* variants, c.1519G>A and/or c.1518T>C, which were detected with unexpectedly high frequency, raising concerns about a possible mis-annotation. Therefore, we aimed to investigate the distribution of these variants in a mono-institutional cohort mainly composed of individuals with a personal and/or family history suggestive of HBOC, and to verify the hypothesis of variant mis-annotation.

## 2. Materials and Methods

### 2.1. Patients

Our laboratory is part of the European Institute of Oncology (IEO) and operates in close collaboration with the Division of Cancer Prevention and Genetics.

For this study, we analyzed 920 individuals with personal or family history suggestive of hereditary cancer predisposition, who were referred to our institute between 2017 and 2021 for a germline genetic test. Informed consent was obtained for all participants. The study was approved by the IEO Institutional Review Board (code UID 4894).

### 2.2. DNA Extraction and Quantification

Genomic DNA was extracted from peripheral blood using a MagCore Super Automated Nucleic Acid Extractor (Diatech, Iesi, Italy) with standard procedures, according to the manufacturer’s instructions. Initial DNA quantification was provided by the MagCore Extractor. For NGS library preparation, DNA was further quantified using the Qubit dsDNA HS Assay kit with the Qubit 3.0 Fluorometer (Life Technologies, Monza, Italy), following the protocol provided by the manufacturer.

### 2.3. Library Preparation and Next-Generation Sequencing (NGS)

All samples were analyzed with the Hereditary Cancer Solution (HCS) CE-IVD multigene panel by Sophia Genetics. We used a customized panel comprising 28 predisposition genes associated with BC, OC, HNPCC (hereditary nonpolyposis colorectal cancer), polyposis, and melanoma, along with *PMS2CL*, a pseudogene of *PMS*2 (for the complete gene list see Table S1). Library preparation was optimized for 200 ng of total gDNA (Qubit quantification), using the enrichment protocol version PM_T1_5.1.5_r2en July 2017. The panel covers the full coding regions as well as 25 bp of non-coding DNA in the exon flanking regions of the included genes. Libraries were quantified using the 4200 TapeStation (Agilent Technologies, Santa Clara, CA, USA) and Qubit 3.0 Fluorometer (Life Technologies, Monza, Italy) and then diluted to 4 nM. Following denaturation, a 10 pM library dilution was loaded for sequencing, with 3% PhiX control, on the Illumina MiSeq system, using the MiSeq V2 Standard reagent Kit 2 × 250 cycles, according to the manufacturer’s instructions. Results were retrieved and analyzed using the dedicated SOPHiA DDM platform (SOPHiA GENETICS, Rolle, Switzerland) by applying the germline pipeline, which accurately detects single-nucleotide variants (SNVs), indels, and copy-number variations (CNVs) [12].

### 2.4. Sanger Sequencing

Validation of the two *BARD1* variants was performed by Sanger sequencing on a second independent blood sample [13].

Conventional PCR was performed in 25 µL reaction volumes containing 100 ng of genomic DNA template; 1X Gold Buffer and MgCl_2_ (Thermo Fisher Scientific, Monza, Italy), primers: 0.2 µM each; 0.2 µM dNTPs (EuroClone, Milano, Italy); 0.2 mM DMSO; 0.5 µL AmpliTaq Gold DNA Polymerase (1.25U; Thermo Fisher Scientific, Italy). PCR amplification was carried out on a Veriti Dx Thermal Cycler (Applied Biosystems, Monza, Italy) as follows: one initial cycle at 96 °C for 7 min; 40 cycles at 94 °C for 30 s, 54 °C for 45 s, 72 °C for 45 s; followed by a final extension at 72 °C for 10 min. PCR products were checked on a 2.0% agarose gel stained with SYBR Safe and purified using the ExoSAP-IT™ PCR Product Cleanup Reagent (Thermo Fisher Scientific, Italy) according to the manufacturer’s instructions. Sequencing reactions were performed according to the BigDye terminator v3.1 protocol (Thermo Fisher Scientific, Italy) and purified with the BigDye XTerminator Purification Kit (Applied Biosystems, Italy). Sequencing products were run on a 3500xL Dx Genetic Analyzer (Applied Biosystems, Italy).

### 2.5. Variant Classification

Genetic variants were classified into five categories following the IARC recommendations [14] and evaluated in accordance with the ACMG/AMP guidelines, with additional gene-specific criteria from ClinGen Expert Panels when available. Variant interpretation was supported by multiple databases and tools, including ClinVar (https://www.ncbi.nlm.nih.gov/clinvar/ accessed on 15 September, 2025), BRCA Exchange (https://brcaexchange.org accessed on 15 September 2025), LOVD (https://www.lovd.nl/ accessed on 15 September 2025), InSiGHT (https://www.insight-database.org accessed on 15 September 2025), and Varsome (https://varsome.com/ accessed on 15 September 2025). In this study, pathogenic and likely pathogenic variants were collectively referred to as PVs.

### 2.6. Analysis of BARD1 Genotype and Allele Frequencies

For each sample, we performed a visual inspection of the reads covering the two *BARD1* variants using the Integrative Genomics Viewer (IGV_2.12.3) software [15] and we calculated the genotype frequencies observed in our cohort.

We used the Allele Frequency Aggregator (ALFA) database from NCBI (https://www.ncbi.nlm.nih.gov/snp/docs/gsr/alfa/ accessed on 15 September 2025) to review the frequencies reported for the mutated and wild-type alleles of these *BARD1* variants in the European population.

## 3. Results

We analyzed 920 individuals of Caucasian ethnicity with a personal or family history suggestive of hereditary cancer predisposition who underwent germline multigene panel testing. The majority of participants were female (856/920 = 93%), with males accounting for only 64/920 (7%). Breast cancer was the most prevalent condition in the cohort (729/920 = 79.2%), with a median age at first diagnosis of 43 years (interquartile range: 37–49). Ovarian cancer was diagnosed in 59/920 (6.4%) patients, with a median age of onset equal to 50 years (interquartile range: 45–58). Notably, 14/920 (1.5%) subjects developed both BC and OC, whereas 146/920 (10.2%) did not develop either BC or OC. Specifically, 94 patients were diagnosed with other cancer types, while 52/920 (5.7%) were healthy subjects (Table S2). Beyond BC and OC, the most frequently observed cancers in the whole cohort included melanoma (29/920 = 3.2%); colorectal cancer (27/920 = 2.9%); endometrial cancer (26/920 = 2.8%); gastric cancer (13/920 = 1.4%) and pancreatic cancer (13/920 = 1.4%).

Out of the 920 individuals, 162 were found to carry at least a PV (162/920 = 17.6%) (Table 1). Specifically, 104 harbored PVs in *BRCA1* or *BRCA2*, and 58 in other genes (17.6%), while 758 did not show any genetic alteration (WT) in the selected genes (82.4%).

Considering only the cohort of 729 patients diagnosed with BC (Table 2), 117 were found to carry at least a PV (117/729 = 16%). In particular, 92 PVs were identified in *BRCA1* and *BRCA2*, and 25 in other genes, while 612 patients showed no genetic alterations (WT) in the selected genes.

Concerning the *BARD1* gene, we identified 159/920 (17.28%) subjects with a pure heterozygous variant c.1518_1519delinsCA (p.Val507Met). This variant is located in the exon 6 of *BARD1*, at position chr2:215632255 (NM_000465.4). At the time of genetic testing, it was classified as a variant of uncertain significance (VUS). Eight out of the 159 carriers were healthy individuals, whereas the remaining 151 patients developed one or more tumors. Table S3 shows the cancer distribution among these 151 patients. As another point, among the 159 subgroups of subjects with the c.1518_1519delinsCA variant, only 27 (27/159 = 17%) carried at least a PV in another gene, mainly *BRCA1*, *BRCA2*, or other BC genes.

Notably, this two-base insertion/deletion variant could also be considered as the simultaneous substitution of two consecutive nucleotides. In the literature, several studies have considered these two substitutions separately (c.1518T>C and c.1519G>A) [7,9,11,16], classifying both as benign.

We determined the different genotypes at these positions in our cohort (reported in Table 3) and their frequencies (reported in Table 4).

A detailed analysis of our data revealed that, in our sample, the c.1519G>A variant (homozygous or heterozygous) was detected exclusively in association with the c.1518T>C, while it is never present in cases harboring the c.1518 WT allele (T). Given our sample size, we can reasonably exclude that this observation results from sampling bias.

The allelic frequency of the c.1519G>A variant in our cohort is 65.48% for the G allele (WT) and 34.51% for the A allele (mutated). Instead, for the c.1518T>C variant, the allelic frequency of the T allele (WT) is 22.11% while that of the C allele (mutated) is 77.88%. We interrogated the NCBI ALFA database and observed that the frequency of the WT allele (T) is around 19%. Consistent with our findings, the data from the ALFA database show that the c.1518T allele—regarded as “wild-type”—is less frequent than the c.1518C allele—regarded as “mutated”. We considered the possibility that, at this genomic position, the reference allele may have been incorrectly annotated, such that the WT allele actually corresponds to the variant currently designated as mutated and vice versa (C as WT and T as mutated). Notably, none of the subjects in our cohort carried c.1519, either heterozygous or homozygous mutated, or the c.1518 WT allele. By contrast, under the hypothesis of a mis-annotation at position c.1518, all possible allelic combinations were observed in our patients (Table 3 and Table 4).

## 4. Discussion

In this study, we analyzed the distribution of the *BARD1* variants c.1518T>C and c.1519G>A in a cohort of 920 individuals with a personal or family history suggestive of a hereditary cancer predisposition syndrome who were referred for germline genetic testing. Our data showed that the allelic frequencies of one of these variants differed from the expected WT frequencies reported in standard reference databases. Specifically, the c.1519G>A variant exhibited an allele frequency of 34.51% for the A allele (considered as the mutated allele), whereas the c.1518T>C variant displayed an even higher mutated allele frequency (77.88%), contrasting with the lower WT frequencies generally reported.

The unexpectedly high prevalence of these variants in our cohort prompted us to explore two main hypotheses regarding their distribution. First, we considered the possibility of population-specific variation. By querying the ALFA database, which compiles allele frequencies across multiple populations, we found that the frequencies observed in our cohort closely matched those reported in ALFA for the European population. This observation suggests that the allelic distributions identified here are not attributable to sampling bias but instead reflect the underlying genetic background of our population.

Second, and perhaps more intriguingly, our findings raise the possibility that the reference genome annotation at position c.1518 may not correspond to the true WT allele in our population. This interpretation is supported by the absence of any cases harboring the c.1519 variant without the concomitant presence of the c.1518 variant, implying a possible linkage or haplotype effect that may be misrepresented in the current reference sequence.

This observation is particularly relevant given that these variants, when considered separately (c.1518T>C and c.1519G>A), have been classified as benign in previous studies [7,9,11,16]. However, the combined dinucleotide substitution (c.1518_1519delinsCA) may be misleading, emphasizing the critical importance of precise variant annotation.

It is also noteworthy that the low frequency of *BARD1* PVs (less than 1%) in HBOC patients contrasts sharply with the much higher frequencies of *BRCA1* and *BRCA2* PVs, reinforcing the view of *BARD1* as a gene conferring a moderate to low risk for breast cancer [17]. Nonetheless, the inclusion of *BARD1* in multigene panels for hereditary breast cancer testing remains justified, as *BARD1* variants may contribute to cancer susceptibility, especially when considered in combination with other genetic or environmental factors [18].

In conclusion, our study underscores the critical importance of accurate variant annotation and population-specific allele frequency data in the interpretation of NGS results. The observed allelic frequencies challenge current reference annotations and emphasize the need to reassess the genetic architecture of this locus in different populations.

Although our sample size was relatively large and comparisons were made with a population-matched public database, some limitations should be acknowledged when considering the apparently higher frequency of the “mutated” alleles. It cannot be conclusively determined that a higher frequency of an allele currently classified as “mutated,” compared with the reference genome, necessarily reflects a misannotation. Variants may be relatively common in specific populations while still exhibiting limited or milder clinical impact. Moreover, even when reference data are geographically matched, subtle subpopulation structures could still influence the observed frequencies.

Future investigations should aim to address these limitations by integrating functional assays, segregation analyses, and large, multiethnic cohorts. Such efforts will be crucial not only for refining variant annotation accuracy but also for improving the clinical interpretation of variants and enhancing precision in risk stratification among individuals and families with hereditary breast and ovarian cancer (HBOC).

## Figures and Tables

**Table 1:** PVs identified in 920 patients.

N. of PVs	Genes
48	*BRCA1*
56	*BRCA2*
23	other BC genes (*ATM*, *BARD1*, *CDH1*, *CHEK2*, *PALB2*, *RAD51C*, *TP53*)
35	other genes
Tot. 162	

**Table 2:** PVs identified in the 729 BC patients.

N. of PVs	Genes
40	*BRCA1*
52	*BRCA2*
20	other BC genes (*ATM*, *BARD1*, *CDH1*, *CHEK2*, *PALB2*, *RAD51C*, *TP53*)
5	other genes
Tot. 117	

**Table 3:** Genotypes and allele frequencies at positions c.1518 and c.1519 as defined by DDM and IGV in our 920 cases.

	Genotype (DDM)	Genotype (IGV)	Allele Frequency (*)	IGV Representation
a	c.1518_1519delinsCA	c.1518C/c.1518C c.1519A/c.1519A	100% 100%	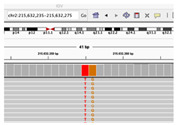
b	c.1518_1519delinsCA c.1518T>C	c.1518C/c.1518C c.1519G/c.1519A	100% 50%	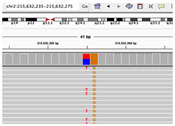
c	c.1518_1519delinsCA	c.1518T/c.1518C c.1519G/c.1519A	50% 50%	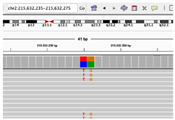
d	c.1518T>C c.1519G>G	c.1518T/c.1518C c.1519G/c.1519G	50% 100%	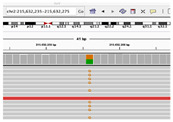
e	c.1518T>C c.1519G>G	c.1518C/c.1518C c.1519G/c.1519G	100% 100%	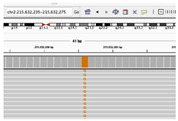
f	c.1518T>T c.1519G>G	c.1518T>T c.1519G>G	100% 100%	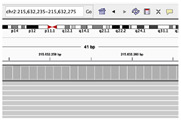

*: allele frequencies refer to IGV genotypes.

**Table 4:** Genotypes, allele frequencies, and sample frequencies at position c.1518 and c.1519 as defined by DDM and IGV in our 920 cases.

Lane in Table 3	Genotype (DDM)	Genotype (IGV)	Allele Frequency (*)	Sample Frequency (%)
a	c.1518_1519delinsCA	c.1518C/c.1518C	100%	102/920 (11.08)
		c.1519A/c.1519A		
b	c.1518_1519delinsCA	c.1518C/c.1518C	100%	272/920 (29.56)
	c.1518T>C	c.1519G/c.1519A	50%	
c	c.1518_1519delinsCA	c.1518T/c.1518C	50%	159/920 (17.28)
		c.1519G/c.1519A		
d	c.1518T>C	c.1518T/c.1518C	50%	162/920 (17.60)
	c.1519G>G	c.1519G/c.1519G	100%	
e	c.1518T>C	c.1518C/c.1518C	100%	182/920 (19.78)
	c.1519G>G	c.1519G/c.1519G		
f	c.1518T>T	c.1518T>T	100%	43/920 (4.67)
	c.1519G>G	c.1519G>G		

*: Allele frequencies refer to IGV genotypes.

## Data Availability

The raw data supporting the conclusions of this article will be made available by the authors on request. Data will be made available on request.

## Supplementary Materials

The following supporting information can be downloaded at: https://www.mdpi.com/article/10.3390/biomedicines13112764/s1, Table S1: List of genes included in the multigene panel. Table S2: Clinical phenotype of the study population. Table S3: Cancer type distribution among the 151 patients carrying the c.1518_1519delinsCA variant.
