# Supplementary material for: Characterization of a Genetic Variant in BARD1 in Subjects Undergoing Germline Testing for Hereditary Tumors
Source: Biomedicines. 2025 Nov 12;13(11):2764. doi: 10.3390/biomedicines13112764 (PMC12650687; doi:10.3390/biomedicines13112764)
Supplement: Supplementary file 1 [file biomedicines-13-02764-s001.zip › biomedicines-3935748-supplementary.pdf]

Supplementary information

Table S1. List of genes included in the multigene panel.

GENE NAME	RefSeq
APC	NM_000038.6
ATM	NM_000051
BARD1	NM_000465
BRCA1	NM_007294
BRCA2	NM_000059
BRIP1	NM_032043
CDH1	NM_004360
CHEK2	NM_007194.4
EPCAM	NM_002354
FAM175A	NM_002354
MLH1	NM_000249
MRE11A	NM_005590
MSH2	NM_000251
MSH6	NM_000179
MUTYH	NM_001128425.2
NBN	NM_002485
PALB2	NM_024675
PIK3CA	NM_006218
PMS2	NM_000535
PTEN	NM_000314
RAD50	NM_005732
RAD51C	NM_058216
RAD51D	NM_002878.4
STK11	NM_000455
TP53	NM_000546
XRCC2	NM_005431
CDKN2A	NM_000077.5
CDK4	NM_000075.4

Table S2. Clinical phenotype of the study population.

	N.	%
Breast cancer	715	77.7%
Ovarian cancer	45	4.9%
Breast and ovarian cancer	14	1.5%
Other cancers*	94	10.2%
Healthy individuals	52	5.7%
	920	100.0%

*excluding breast cancer and ovarian cancer

Table S3. Cancer type distribution among the 151 patients carrying the c.1518_1519delinsCA variant.

	**Patients***	**%**
Breast cancer	119	74.8%
Ovarian cancer	11	6.9%
Melanoma	8	5.0%
Colorectal cancer	7	4.4%
Gastric cancer	6	3.8%
Endometrial cancer	5	3.1%
Thyroid cancer	2	1.3%
Renal cancer	1	0.6%
Sarcoma	1	0.6%
Bladder cancer	1	0.6%
Other cancers	5	3.1%

*The sum of the absolute values does not equal 151 (and the sum of the percentages does not add up to 100%) due to the fact that some patients developed multiple tumors.
